# Revealing the spatial shifting pattern of COVID-19 pandemic in the United States

**DOI:** 10.1038/s41598-021-87902-8

**Published:** 2021-04-19

**Authors:** Di Zhu, Xinyue Ye, Steven Manson

**Affiliations:** 1 Department of Geography, Environment and Society, University of Minnesota, Twin Cities, USA; 2 Department of Landscape Architecture and Urban Planning, Texas A&M University, College Station, USA; 3 Beijing Key Lab of Spatial Information Integration and Its Applications, Peking University, Beijing, China

**Keywords:** Environmental social sciences, Socioeconomic scenarios, Sustainability

## Abstract

We describe the use of network modeling to capture the shifting spatiotemporal nature of the COVID-19 pandemic. The most common approach to tracking COVID-19 cases over time and space is to examine a series of maps that provide snapshots of the pandemic. A series of snapshots can convey the spatial nature of cases but often rely on subjective interpretation to assess how the pandemic is shifting in severity through time and space. We present a novel application of network optimization to a standard series of snapshots to better reveal how the spatial centres of the pandemic shifted spatially over time in the mainland United States under a mix of interventions. We find a global spatial shifting pattern with stable pandemic centres and both local and long-range interactions. Metrics derived from the daily nature of spatial shifts are introduced to help evaluate the pandemic situation at regional scales. We also highlight the value of reviewing pandemics through local spatial shifts to uncover dynamic relationships among and within regions, such as spillover and concentration among states. This new way of examining the COVID-19 pandemic in terms of network-based spatial shifts offers new story lines in understanding how the pandemic spread in geography.

## Introduction

The COVID-19 pandemic poses a global threat to human health and socioeconomic well being. The rapid escalation of the epidemic in the United States (U.S.) offers a compelling case study in tracking the spatiotemporal nature of disease spread. The number of total confirmed cases reached 7 million on September 24, 2020, a vast increase since the first domestic case was reported on January 21, 2020^1^. One of the most common approaches to tracking COVID-19 dynamics is through regular snapshots in the form of choropleth maps, or where the number of cases are mapped by administrative units such as states or counties^2^. One may gain a sense of dynamics - change over time - by manually toggling back and forth through the maps or by developing a change map, where rates or differences are calculated on a per-region basis between two snapshots^3,4^. While such mapping is integral to understanding and responding to the pandemic, there remains a subjective element when the viewer flips back and forth between maps or must interpret change between two fixed dates among many. It can be difficult to assess the impacts of mandates such as wearing masks, social distancing and lockdowns, that have been proved to be effective to help reduce the risk of disease transmission^5–7^, alongside mobility restrictions and greater geographic distancing^8–12^. These interventions operate across scales (from local to regional to national) and can have second-order spatial interactions^13–15^ in the sense that a change in one locality will take time to propagate through space and time to other localities^16,17^. Relying on static snapshots via choropleth maps can make it difficult to fully capture the change over time in severity for given locations or to interpret these second-order impacts.

We offer a new approach to understanding the spatiotemporal processes of COVID-19—and more generally, dynamic processes over space—by capturing the shifting centres of the pandemic over time. We extend existing research on network modeling^18^ to infer the shifting spatial patterns of the COVID-19 pandemic among the contiguous mainland U.S. states (i.e., excluding Alaska and Hawaii). Importantly, this method can leverage existing data, namely the sequential snapshots of COVID-19 confirmed cases that are used to develop standard choropleth maps. This approach uses these simple data—total confirmed case numbers by spatial unit such as country or state—in pairs of snapshots and treats them as constraints for inferring spatial contagion processes. In particular, we use linear programming in a spatial network optimization framework to infer the spatially shifting intensity of cases between snapshots. By stringing together a series of snapshots it becomes possible to chart the course of the pandemic over time and space. The strategy for calculating spatial shifts between snapshots is analogous to solving a minimum-cost flow problem in network optimization^19,20^, which aims at finding the optimal flow (shift) configuration in a network that is subject to the variations (new confirmed cases) at all nodes (states) (*Supplementary Information*, Note 1). The unit cost for pandemic shifts is modelled as a combined effect of both geographical and social distancing, where the gravity model with distance decay^21,22^ is adopted to quantify the geographical distancing among states and the human movements derived from geotagged Twitter data are used to characterize the inter-state mobility restrictions.

This work offers a new way to describe and understand the COVID-19 pandemic by giving insight into the shifting centres and spread across the country. It builds on, complements and expands the common approach of snapshots and choropleth maps. While the focus is on the COVID-19 pandemic of the U.S. in this research, our approach holds promise for other epidemiological scenarios and complex spatiotemporal human-environment interactions more broadly at different geographical scales, especially when we only have sequential snapshots of geospatial distribution data and the unknown second-order spatial processes are to be inferred or predicted.

## Results

We chose a series of epidemic snapshots and attendant periods for this analysis based on a combination of key events collected from CNN online news^23^ and cumulative confirmed cases reported by the New York Times^1^ from January through August 2020 (see *Methods* for more information on the data). For each of these phases, we use network modeling to construct a flow map of the inferred spatial shifts at the state-level. While the number of confirmed cases consistently increased over time, we notice a global pattern with stable pandemic centres characterized by both local and long-range spatial shifts. We then examine specific metrics that can help quantitatively evaluate the evolving nature of the pandemic. On the other hand, the locally shifting patterns give insight into dynamic spatial relationships at local and regional scales, such as spillover among states or concentration of cases among clusters of states.

### Spatiotemporal shifts in COVID-19 cases

**Figure 1:**
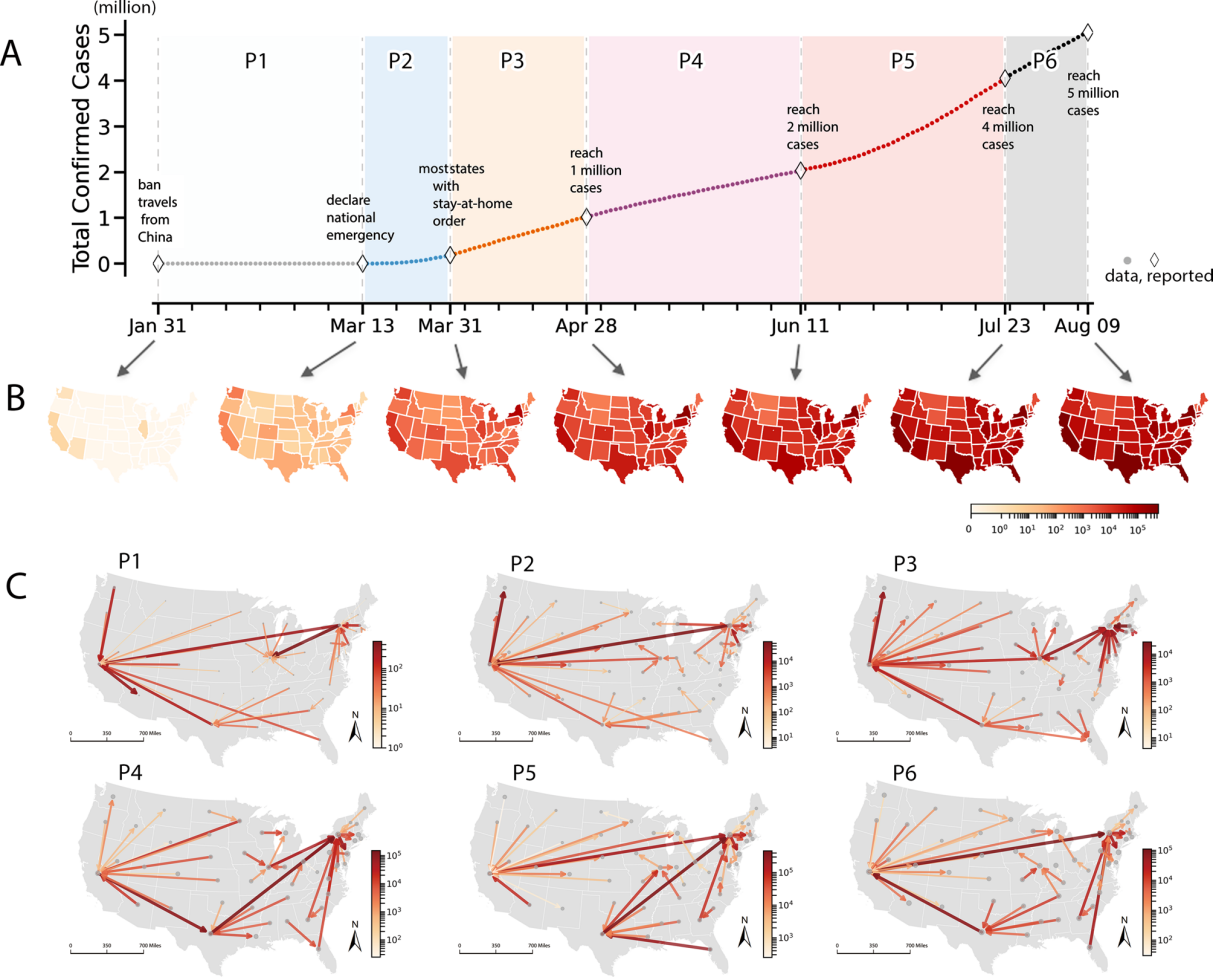
Six phases of the COVID-19 pandemic and their corresponding inferred spatial shifting patterns in the United States. (**A**) Timeline of the total confirmed cases (*CCs*) in the U.S. from Jan. 31 to Aug. 9, 2020^1^. The timeline is divided into six phases: P1: Jan. 31–Mar. 13, since all travel from China was banned until the government declared national emergency; P2: Mar. 13–Mar. 31, state stay-at-home orders become prevalent; P3: Mar. 31–Apr. 28, 1 million cases; P4: Apr. 28–Jun. 11, 2 million cases; P5: Jun. 11–Jul. 23, 4 million cases; P6: Jul. 23–Aug. 09, 5 million cases. (**B**) Seven epidemic snapshots of *CCs* at the state-level. For each day in the timeline, the reported data from^1^ was used to make a snapshot of *CCs*. The variation between two rescaled snapshots is used to infer the spatial shifts. (**C**) Flow maps of the inferred spatial shifts for the six phases. Shifts are drawn as arrows among states indicating where they come from and where they shift to in terms of case counts. The colour and width of all arrows is linearly mapped according to the intensity of shifts.

We examine seven snapshots and six periods along the timeline from January 31, 2020 to August 09, 2020. The first three snapshots are selected based on the first confirmed case, declaration of the national pandemic emergency, and widespread adoption of stay-at-home orders, respectively. These policies in the early stages of the pandemic explicitly restricted international and domestic travel^23^. The remaining four snapshots are based on thresholds of cumulative confirmed cases at one, two, four and five million, respectively. Figure 1A shows the timeline from Jan. 31 to Aug. 9 as six pandemic phases that correspond to key moments in the pandemic in the U.S. (see *Supplementary Information*, Tab. S1 for details). The start date is chosen as Jan. 31 because on that date, the U.S. banned travel to the nation from China and the other countries. This date is doubly important because we can treat the mainland U.S as fairly self-contained system in which each period is capturing internal spread as the primary spatial process driving spatiotemporal shifts in COVID-19 and attendant variation in observed confirmed cases. Phase 1 (P1) can be considered as a period when the case count was not severe but clearly there was COVID-19 spread in the absence of major public health interventions. Phase 2 (P2) starts on Mar. 13, the date when the federal government declared a national emergency and extends until Mar. 31, when most states implemented stay-at-home mandates designed to stem disease transmission^12,24^. Phase 3 (P3) is defined by when inter-state mobility was the lowest according to twitter data (see *Supplementary Information*, Fig. S5) and extends to when the number of confirmed case reached 1 million on Apr. 28. Subsequent phases use the same rational of major milestones, where Phase 4 (P4), Phase 5 (P5) and Phase 6 (P6) are defined by the dramatic rise in cases from 1 million to 2 million to 5 million cases respectively.

For each snapshot $S^{(t)}$ at time *t* in Fig. 1B, the total confirmed case data are formalized as a vector $D^{(t)}=[d_1^{(t)}, d_2^{(t)}, \dots , d_n^{(t)}]$, where $n=48$ is the number of states, and $d_i^{(t)}$ denotes the data of state *i* on time point *t*. The variation of confirmed cases within each state *i* during the $t^{th}$ phase is then calculated as $\Delta cc_i^{(t)}=d_i^{(t+1)}-d_i^{(t)'}$. Here, $d_i^{(t)'}=d_i^{(t)}\frac{\sum _i d_i^{(t+1)}}{\sum _i d_i^{(t)}}$ is the rescaled number of cases ensuring the total number of confirmed cases (*CCs*) remains unchanged between $S^{(t)}$ and $S^{(t+1)}$, as discussed in *Methods*.

To model the possibility of spatial shifts between state *i* and *j* during the $t^{th}$ phase, we adopt both geographical distancing and social distancing constraints to define the unit cost, i.e., $c_{ij}^{(t)}=k(\frac{d_{ij}^\beta }{A_iA_j} )(\frac{1}{log_{10}(m_{ij}^{(t)}+\delta )} )$. The first term $d_{ij}^{\beta }/{A_iA_j}$ is an inverse form of the prevailing gravity-law^25,26^ in modelling spatial interactions^21,22,27^, indicating that $c_{ij}$ increases with a larger geographical displacement $d_{ij}$, while decreases as the states’ attraction product $A_iA_j$ are stronger. The second term $1/(log_{10}(m_{ij}^{(t)}+\delta ))$, on the other hand, characterizes the dynamic social distancing reflected in the number of inter-state Twitter movements $m_{ij}^{(t)}$ from state *i* to *j* during the $t^{th}$ phase. This cost definition combines a mix of interventions from geographical segregation and human mobility restrictions. It indicates that the unit cost of spatial shifts increases at a sub-linear rate with distance when $\beta < 1$, which is consistent with literature in regional studies and spatial interaction modeling (see *Methods* and *Supplementary Information*, Note 2 for details).

We then construct a network optimization task that incorporates all costs $C\in {\mathbb {R}}^{n\times n}$ and case variations $\Delta CCs\in {\mathbb {R}}^{n\times 1}$ into a linear program to calculate the optimal spatial shifts $X \in {\mathbb {R}}^{n\times n}$ that minimize shifts’ total cost during each pandemic phase (see *Methods* and *Supplementary Information*, Note 1 for details). Coefficients *k* and $\delta$ do not affect the intensities of inferred shifts. We present the results using census resident population as the primary source of attraction, $k=10^8$, $\delta =1$, and a distance decay parameter $\beta =0.8$. The inferred spatial shifts *X* are plotted as flow maps in Fig. 1C, where each aggregated pair-wise flow $x_{ij}\in X$ is drawn as an arrow coming from state *i* and shifting into state *j*. The colours and widths of all arrows are linearly mapped according to the intensity of shifts $log_{10}(X)$.

In Fig. 1C, we notice a stable spatial shifting pattern of pandemic centres throughout the six phases. As the total *CCs* consistently increases, major population centers including California (CA), New York (NY), Texas (TX), Illinois (IL) remain as the local concentration centres in the network. We observe long-range, strong shifts between states such as NY and CA (P1, P2, P5, P6), NY and IL (P1, P3, P4), CA and TX (P1, P3, P4, P6), TX and NY (P4, P5). These dominant states exhibit in-shifts from distant states and also shift out to nearby states with fewer COVID-19 cases across phases. Seeing these shifting patterns is helpful in seeing how states with larger economies and populations tend to have stronger spatial shifts in the system (*Supplementary Information*, Fig. S6). Specifically, CA, NY, IL and TX exchange major shifts as well as movement of cases into surrounding states. Meanwhile, some states with nonnegligible outbreaks during P4 and P5, such as New Jersey (NJ), Massachusetts (MA) and Georgia (GA), exhibit a pattern of first shifting out and then receiving in-shifts. We can also see how GA and Florida (FL) emerge in the flow maps of P4, P5 and P6 with strong out-shifts, implying potential outbreaks in later phases.

The intensity and distance of spatial shifts indicate how the pandemic develops across phases. Statistically speaking, the sum intensity of pandemic shifts reaches its peak in P5: $2.3\times 10^3$ (P1), $1.56\times 10^5$ (P2), $1.83\times 10^5$ (P3), $6.63 \times 10^5$ (P4), $2.00 \times 10^6$ (P5), $4.77\times 10^5$ (P6). The mean distance (*km*) of non-zero shifts first decreases and then increases: 868.94 (P1), 848.57 (P2), 789.59 (P3), 776.40 (P4), 828.57 (P5), 831.60 (P6) (*Supplementary Information*, Fig. S7). While this is not an epidemiological study, these numbers seem to correspond with how, during P2 and P3, people followed stay-at-home orders and attendant rules around social-distancing, resulting in more short-range shifts. In contrast, in P4 and P5, these mandates started to lose efficacy due to complex socioeconomic reasons such as COVID fatigue, where people grow tired of rigidly adhering to public health guidance. The intensity and distance of shifts thus increase dramatically during P4 and P5, indicating a surge in COVID-19 cases and a follow-up new wave of pandemic outbreaks. This said, knowledge of the COVID-19 pandemic is growing by the day and there could be other reasons why we see these shifts. Nonetheless, our approach offers a new way to see the shifting spatiotemporal nature of the disease.

In *Supplementary Information*, Fig. S4, we visualize the aggregated twitter movements across states. The location of each active twitter user is calculated as the mean centre of all posted tweets on a daily basis. Then the spatiotemporal information is aggregated according to the time periods and state-level administrative boundaries to show how people actually travel among U.S. states^6,14^. *Supplementary Information*, Fig. S5 indicates that the inter-state movements experienced an evident reduction during P1, P2 and reached the lowest in P3, while started to increase again after that. By examining the twitter movements together with the spatial shifts shown in Fig. 1B, we observe that even though human mobility declined during P1, P2 and P3, larger patterns remained similar and the total intensity of shifts still increased. The major difference in P3, compared to P1 and P2, is that IL became a junction state that bridges the shift between CA and NY, showing more critical role of the central United States. After P3, we observe a stable pattern with three major pivots in the network, i.e., CA in the west, TX and IL in the middle, NY, GA and FL in the east.

In the following section, we focus on the optimal spatial shifts shown in Fig. 1C. Both the global shifting pattern and local shifting patterns are analysed to further evaluate how the related metrics of spatial shifts computed from the pandemic snapshots can be used as indicators to help understanding the development of COVID-19 pandemic in the U.S.

### Daily metrics of shifts at regional scales

Looking at daily shifts among states can contextualize the broad shifts in intensity across phases seen above. Three metrics in particular are useful: daily shift among states; observed variation of confirmed cases with respect to in-shifts and out-shifts; and daily costs of shifts as a measure of severity of the pandemic in states. These metrics give insight into the nature of how the pandemic plays out over time.

**Figure 2:**
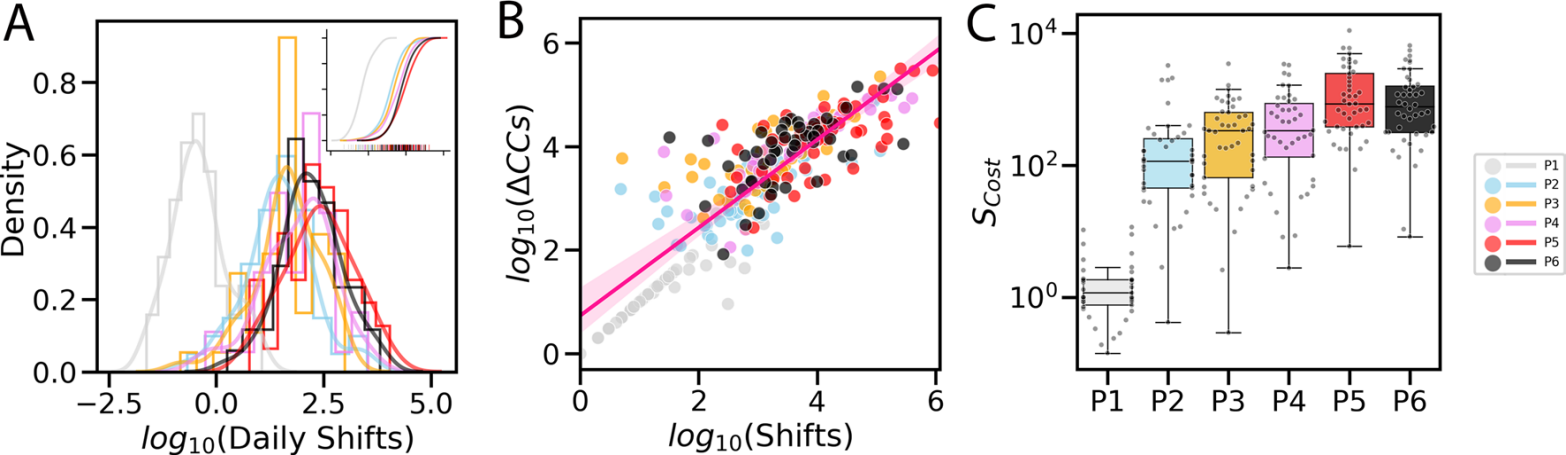
Metrics of spatial shifts quantitatively depict the pandemic. We use six colours to differentiate the data in the six phases. (**A**) Probability distributions of daily shift intensities. (**B**) Scatter plot of shift intensities and the new confirmed cases shows a significant positive correlation: Pearson $R=$ 0.86, $p\approx$ 0. (**C**) Box plot of the daily cost of shifts illustrates the dynamic severity and complexity of the pandemic across phases.

First, we define the daily pandemic shift between state *i* and *j* in the $t^{th}$ phase as $x^{*(t)}_{ij}=x^{(t)}_{ij}/\Gamma ^{(t)}$, where $x^{(t)}_{ij}$ is the total shifts between state *i* and *j* in the $t^{th}$ phase, and $\Gamma ^{(t)}$ is the number of days in the $t^{th}$ phase. Daily shifts denote how the pandemic is shifting via a daily average during a period. Compared to the total shifts, daily shifts are independent of the duration of a phase and offer a more intuitive take on evaluating the severity of the pandemic. Figure 2A shows the histogram distributions with kernel density estimation (KDE) of daily shift intensities $log_{10}(x^{*})$ for six phases. P1 has the lowest daily shifts, with only a few states with confirmed cases during the early stage of spreading. centres such as CA, IL, NY and TX are mostly attracting in-shifts from nearby states in P1. However, we observe the roughly same distributions in the other five phases, which reconfirms our findings in Fig. 1C. Despite the existence of epidemic prevention measures, the overall intensities of daily shifts are stable. On the upper-right subplot of Fig. 2A, we illustrate the cumulative distribution of the daily shifts. Compared to the sharp outbreak between P1 and P2, only mild increases in daily shifts can be found in the later phases. We find that there were more strong daily shifts in P5 than in P6, indicating a slight slow down in how the pandemic was spreading.

Second, we evaluate the relationships between the observed variation of confirmed cases $\Delta CCs$ at each state (*Supplementary Information*, Fig. S3) with respect to three indices: the total intensity of in-shifts and out-shifts ($log_{10}$(Shifts)), intensity of out-shifts ($log_{10}$(Out-Shifts)) and intensity of in-shifts ($log_{10}$(In-Shifts)). In Fig. 2B, we observe a significant positive correlation between $log_{10}(\Delta CCs)$ and $log_{10}$(Shifts) (Pearson: $R=0.86$, $p\approx 0$). The reported slope of ordinary least squares (OLS) is 0.851, meaning that $\Delta CCs$ increase at a sublinear rate with shift intensities. As shown in *Supplementary Information*, Figs. S8A and S8B, we also notice significant positive correlations between $log_{10}(\Delta CCs)$ and $log_{10}$(Out-Shifts) (Pearson: $R=0.68$, $p\approx 0$), $log_{10}(\Delta CCs)$ and $log_{10}$(In-Shifts) (Pearson: $R=0.42$, $p\approx 0$). Furthermore, we consider cross-phase relationships between states’ daily shifts in a previous phase and their daily $\Delta CCs$ in the following phase as shown in *Supplementary Information*, Fig. S8C. Again, we notice a significant positive correlation (Pearson: $R=0.72$, $p\approx 0$; Spearman: $R=0.75$, $p\approx 0$), showing that we could use the inferred spatial shifts from historical snapshots to predict outbreaks in the future. These strong relationships between pandemic shifts and the new confirmed cases contextualize existing explanations in^28^, where population flows were used to predict COVID-19 distributions in Wuhan, China. Our findings imply that for a pair of two states with significant population flows in the previous period, the corresponding pandemic shift can be strong, which would lead to a higher possibility of outbreak in the near future.

As a third metric, we introduce the daily cost of shifts $S_{cost}(ij)=x^{*}_{ij}*c_{ij}$ as a hybrid indicator to measure the severity and complexity of the pandemic with respect to how strong the shifts are ($x^{*}_{ij}$) and how difficult it is for the shifts to occur ($c_{ij}$). In Fig. 2C, we show a box plot of all $x^{*}_{ij}*c_{ij}$ in different phases, the scatter points are adjusted so that they do not overlap. The sums of $S_{cost}$ are: 86.88 (P1), 15057.56 (P2), 21326.40 (P3), 29385.62 (P4), 77123.01 (P5) and 60393.67 (P6). The medians $\bar{S}_{cost}$ are: 1.17 (P1), 117.0 (P2), 335.47 (P3), 339.65 (P4), 857.95 (P5) and 773.87 (P6). The standard deviations $\sigma (S_{cost})$ are: 2.63 (P1), 672.53 (P2), 606.49 (P3), 833.19 (P4), 2135.68 (P5), 1536.03 (P6). It is immediately clear that P5 is when the pandemic becomes out of control compared to the other phases. The sums and medians of ${S}_{cost}$ in P5 rise higher than P4, indicating a much more severe situation. At the same time, $\sigma (S_{cost})$ also reaches its highest value in P5, meaning that the spatial shifts during P5 are more complex, with higher diversity in shift intensities and costs. After P5, we observe a slight slowdown in P6. These results are important as they point out the fact that despite the overall situation remaining stable in P5 and P6, the national situation became noticeably worse after P4. Here we only analyse the data till Aug. 09, 2020, but given the timely updating of epidemic snapshots, future work could look at the pandemic for a longer term using similar metrics.

### Spatial shifts at state and local scales

Apart from looking at national-scale statistical metrics, the approaches developed here can also shed light on local and regional dynamics. Doing so may give insight into how the local pandemic centres are moving, how well state reaction control measures are working, or a better understanding of where potential outbreaks in other high-risk regions may occur.

**Figure 3:**
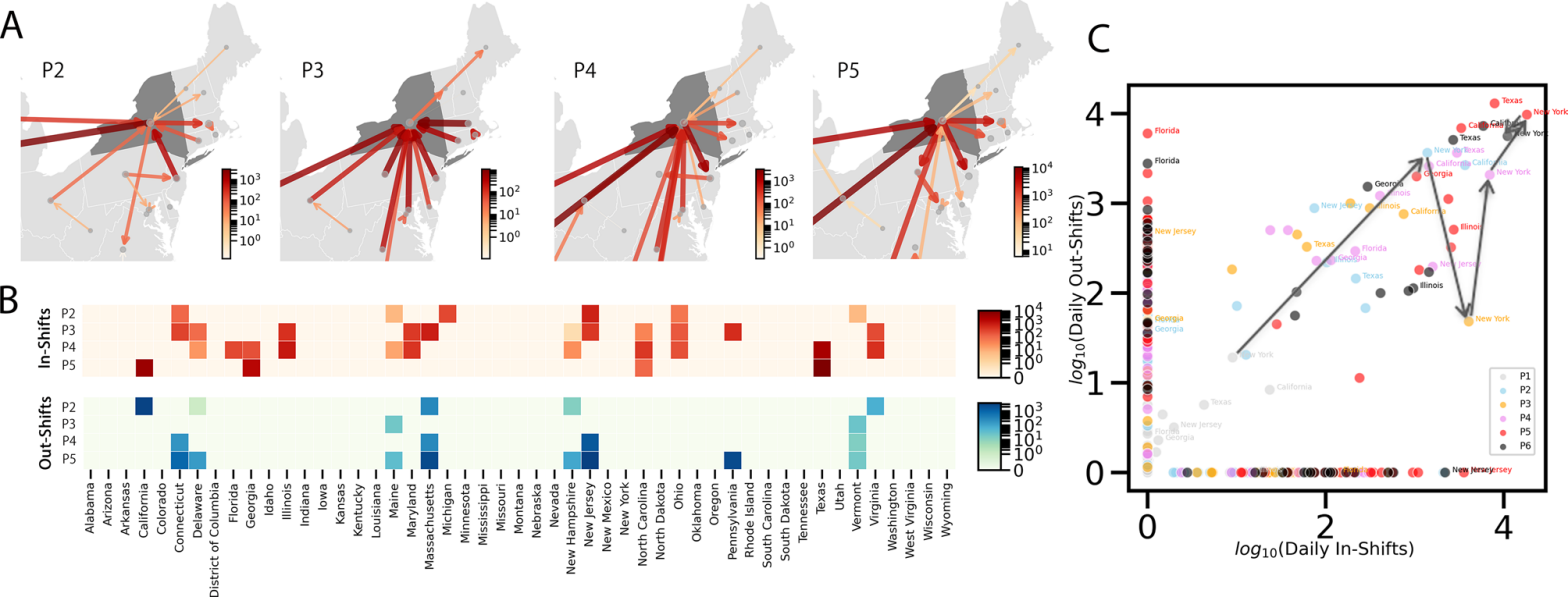
Local spatial shifting patterns indicate the changing situation of a state. (**A**) Flow maps of local spatial shifts around New York (NY) in P2, P3, P4 and P5. (**B**) Heat maps of the shift matrix with NY as the origin or destination in the four phases. (**C**) Temporal scatter plot of the daily in-shift and out-shift intensities. Arrows are used to connect all data points of NY in a temporal sequential order.

Take as an example the spatial shifts from P2 to P5 in New York (NY). The flow maps of local spatial shifts around NY are illustrated in Fig. 3A, where the coloring of arrows is the same as that of Fig. 1C. Figure 3B is a heat map of the shift matrix clarifying where the in-shifts to NY are coming from and where the out-shifts from NY are going to in each phase. Overall, NY transitions from being a “black hole” to a “volcano” in its relationships with other states. During P2, NY started to show the potential for becoming the hub of the pandemic in the northeast U.S., with out-shifts to CA, MA, Virginia (VA), and in-shifts from NJ, Connecticut (CT) and Michigan (MI). In-shifts and out-shifts are roughly balanced in intensity and most shifts are within the Northeast except for the out-shift to CA. In P3, we see a local “black hole” effect in the sense that there are far more in-shifts than out-shifts from almost all nearby states, including CT, Delaware (DE), Maryland (MD), MA, New Hampshire (NH), NJ, North Carolina (NC), Ohio (OH), Pennsylvania (PA) and VA, while out-shifts only occur for Maine (ME) and Vermont (VT). Again, while this is not an epidemiological study, anecdotally, this local concentration in NY occurred during the stay-at-home order and when the median travel distances of people were decreasing for all states after the order^6^. P3 is also when NY was experiencing its fastest increase of case number in April (*Supplementary Information*, Fig. S2). This significant in-shift concentration is an indicator of an ongoing pandemic outbreak. In P4, NY started again to show out-shifts to nearby states, especially NJ, which exhibited a delayed rising curve compared with that of NY (*Supplementary Information*, Fig. S2). Strong in-shifts were coming from farther states such as FL, GA, IL, NC, TX and VA. This is a sign of NY becoming more influential regarding spatial shifts and having a bigger impact on nearby states via a spatial spillover effect^29,30^. In P5, we can see an “active volcano” effect when NY was receiving strong in-shifts from distant states, including CA, GA, TX and NC, and out-shifting to its nearby states in northeast America.

The shifting nature of the pandemic at the state-level can also be analysed using a temporal scatter plot (Fig. 3C). Each point denotes the intensity of daily in-shift and daily out-shift of a state for each phase. We may use arrows to connect all points of a state over time to check how the locally shifting pattern is changing across phases. As shown in Fig. 3C, NY saw a significant drop in out-shifts and a rise of in-shifts between P2 and P3, which indicates a local concentration within the state. After P3, NY gradually returned to a situation marked by increasing out-shifts corresponding to local spillover from NY to nearby states. In sum, state-scale spatial shifts are useful in examining local and regional dynamics.

## Discussion

We adopt a network optimization approach to model spatial shifts over time of the COVID-19 pandemic in the U.S. We visualize these shifts via geographic flow maps to show how the disease centers move over space as the pandemic progresses. This view of the pandemic - based on standard data sets - grants insight into national and regional dynamics. Metrics derived from the daily nature of these spatial shifts can help depict the global pandemic situation in a quantitative way. Finally, the network optimization approach can be applied at regional scales to explore shifting spatiotemporal patterns and underlying relationships among states during the pandemic.

This work offers several advances in the modeling of disease and other spatiotemporal phenomena. First, it offers a new way to track the COVID-19 pandemic from the perspective of spatial shifts that goes beyond commonly-used spatial distribution maps by offering a way to infer spatial interactions over time. Second, by virtue of introducing a temporal element, daily metrics of spatial shifts can be used to analyse the pandemic in new ways, such as the intensity of daily shifts, the association between shifts and new confirmed cases, as well as the total cost of daily shifts. Third, this approach offers a way to capture local shifts in timing and spatial patterning that can give insight into complex dynamics such as spillover and concentration in a complex process like disease progression. In sum, this work offers a new and potentially powerful geospatial tool to review, understand and predict the ongoing pandemic and more broadly, other dynamic spatial processes. Future works are invited to extend our results for other interested regions, or to conduct similar analysis at other geographical scales (e.g., worldwide, continental or provincial) for more shifting knowledge of the pandemic. Also, the latest epidemic snapshots can be used in practice when certain public policies or vaccine interventions are to be evaluated regarding their timely effects on the pandemic.

Our research is subject to several limitations. One, this work is based on the state-level reported case number of confirmed patients, which does not characterize the actual number of cases or severity of COVID-19. Other epidemiologically important attributes such as the generation time, infection rate and incubation period^17^ could be integrated into this analysis to provide a more comprehensive picture of the pandemic’s spatial and temporal shifts. Two, a basic assumption of network optimization is that the nation can be treated as a closed system. While the country has seen severely curtailed international travel, future work would want to include data that captures the impact of external sources of cases. Three, the modelling of shift costs could be improved. The spatial heterogeneity of distance decay parameter $\beta$ is not considered in this work. A higher $\beta$ has the effect or reducing shifts while a lower $\beta$ denotes greater capacity for long-distance shifts. Future work would expect to integrate data-driven techniques such as artificial intelligence and machine learning in calibrating the variation of $\beta$ in space. Four, different human mobility data sets^31,32^ other than Twitter could lead to differing characterization of mobility restrictions than those used here. In *Supplementary Information*, Note 2, we discuss the potential usage of other cost models to modify the spatial shifts; for example, this approach is flexible enough to incorporate geospatial knowledge on populations and their propensity to move derived from census and demographic data^33^ into the cost modelling.

## Methods

### Calculating the optimal spatial shifts between snapshots

Considering a study area with a set ($\mathbf{N}$) of *n* spatial units (states in this work), we formalize the data of cumulative COVID-19 confirmed cases in two consecutive epidemic snapshots, $S^{(t_1)}$ at time $t_1$ and $S^{(t_2)}$ at time $t_2$ ($t_1$ earlier than $t_2$) as:

1 $$\begin{aligned} \begin{aligned} D^{(t_1)}=[d_1^{(t_1)},d_2^{(t_1)},\ldots ,d_i^{(t_1)},\ldots ,d_{n-1}^{(t_1)},d_n^{(t_1)}]\ \\ D^{(t_2)}=[d_1^{(t_2)},d_2^{(t_2)},\ldots ,d_i^{(t_2)},\ldots ,d_{n-1}^{(t_2)},d_n^{(t_2)}], \end{aligned} \end{aligned}$$

where $d_i^{(t_1)}$ and $d_i^{(t_2)}$ are the reported total confirmed cases in state $n_i\in \mathbf{N}$ by time $t_1$ and $t_2$, respectively. Since $d_i^{(t_1)}<d_i^{(t_2)}$ applies for all states at all time, we define $d_i^{(t_1)'}=d_i^{(t_1)}\sum _i d_i^{(t_2)}/\sum _i d_i^{(t_1)}$ as the rescaled number of $d_i^{(t_1)}$. The variation of confirmed cases at state $n_i$ is $\Delta cc_i=d_i^{(t_2)}-d_i^{(t_1)'}$, ensuring a closed and static regional system with $\sum _i d_i^{(t_1)'}=\sum _i d_i^{(t_2)}$. We use cost matrix $C\in {\mathbb {R}}^{n\times n}$ to describe the shifts’ costs between $t_1$ and $t_2$, where $c_{ij}\in C$ is the unit cost of the shift from state $n_i$ to $n_j$. Also, we assume a fully-connected shift matrix $X \in {\mathbb {R}}^{n\times n}$, where $x_{ij}\in X$ is the spatial shift variable from state $n_i$ to $n_j$ to be calculated. Following an existing strategy named *Inferring Interactions from Distribution Snapshots* (IIDS)^18^, the spatial optimization tasks of inferring spatial shifts are constructed in a linear program as follows:

2 $$\begin{aligned} \begin{aligned} minimize&\qquad C^T \times X\\ subject\ to&\qquad -\sum _{j \in \mathbf{N} }x_{ij}+\sum _{j \in \mathbf{N} }x_{ji}=\Delta cc_{i},\quad \forall i \in \mathbf{N} \\&\qquad x_{ij} \in {\mathbb {R}}, \quad x_{ij}\ge 0 \quad \forall i,j \in \mathbf{N} \end{aligned} \end{aligned}$$

The interpretation of inferred *X* is the shifts of pandemic’s spatial centres with respect to the number of COVID-19 confirmed cases. In *Supplementary Information*, Note 1, we describe step by step on how to compute the optimal solution for *X* in Eq. (2) using a synthetic simple example.

### Modelling the cost of spatial shifts

The cost matrix *C* denotes the possibilities of spatial shifts to occur among states. In order to model the heterogeneity of cost in space, we consider both geographical distance decay and social distancing constraints in a unit shift cost from state $n_i$ to $n_j$:

3 $$\begin{aligned} \begin{aligned} c_{ij}&\propto \frac{G_{ij}}{T_{ij}}. \end{aligned} \end{aligned}$$

Here, $G_{ij}=k\frac{d_{ij}^\beta }{A_iA_j}$ is a term derived from the gravity-law in spatial interaction models^25,27^ where the distance *d* and state’s attraction *A* are considered to capture the effect of geographical distancing. Whilst $T_{ij}=log_{10}(m_{ij}+\delta )$ is a social distancing term calculated using the aggregated twitter movements $m_{ij}$ from state $n_i$ to $n_j$, where logarithmic transformation is applied to $m_{i,j}$ to reduce the skewness of twitter data distribution and $\delta$ is a threshold parameter to avoid zero value of $m_{i,j}$. More discussions on the modelling of spatial shift costs can be found in *Supplementary Information*, Note 2.

### Data collection and preprocessing

First, the COVID-19 data was collected from the New York Times, based on reports from state and local health agencies^1^. The reported data of cumulative counts of confirmed coronavirus cases can be used to draw epidemic snapshot maps at the state or county level over time. The raw data begins with the first reported coronavirus case in Washington State on Jan. 21, 2020 and is updating to date. The COVID-19 data is available for free download at https://github.com/nytimes/covid-19-data. The timeline of COVID-19 outbreak was mainly collected from CNN health news^23^. COVID-19 related fast facts were further utilized to determine the six phases in our study (see *Supplementary Information*, Tab. S1 for detailed descriptions). We plot the temporal curves of the total confirmed case for several selected states in *Supplementary Information*, Fig. S2 to show the variation of COVID-19 cases across states. Whilst in *Supplementary Information*, Fig. S3, we illustrate the rank-size distribution of new confirmed cases in each pandemic phase to depict the changing spatial distributions of the data along the timeline. Second, the Twitter movements were derived from the individual geotagged Twitter data. We have collected about 200 million geotagged tweets during the study period, from over 2.9 million unique Twitter users in the U.S. using the official Twitter Streaming Application Programming Interface (API)^31^. Further, we computed a twitter movement matrix that contains the aggregated movement frequency from one state (origin) to another (destination) during each phase. The location of each user is calculated as the mean centre of all posted tweets on a daily basis. The aggregated Twitter movements are visualized and analysed in *Supplementary Information*, Figs. S4,S5. Third, the Gross Domestic Products (GDP) by state in 2019 were collected from the U.S. Bureau of Economic Analysis (BEA) to support the correlation analysis in *Supplementary Information*, Fig. S6. The state-level resident populations reported by the government census on Jul. 1, 2019 were used as the proxy of state attractions in the gravity-based modelling of shift costs. The GDP and population data are publicly available at https://www.bea.gov and https://data.census.gov/cedsci, respectively.

## Supplementary information

Supplementary Informations.

## Data Availability

All code and data needed to replicate our results and conduct the map visualization would be available at https://github.com/dizhu-gis/CovIDSpatialShifts once the paper is published.
