# Supplementary material for: Revealing the spatial shifting pattern of COVID-19 pandemic in the United States
Source: Sci Rep. 2021 Apr 19;11:8396. doi: 10.1038/s41598-021-87902-8 (PMC8055907; doi:10.1038/s41598-021-87902-8)
Supplement: Supplementary file 1 — Supplementary Informations. [file 41598_2021_87902_MOESM1_ESM.pdf]

Supplementary Information: Revealing the Spatial Shifting Pattern of COVID-19 Pandemic in the United States

Di Zhu[1,3,*], Xinyue Ye[2], and Steven Manson[1]

[1]Department of Geography, Environment and Society, University of Minnesota, Twin Cities.
[2]Department of Landscape Architecture and Urban Planning, Texas A&M University.
[3]Beijing Key Lab of Spatial Information Integration and Its Applications, Peking University.
[*]dizhu@umn.edu

ABSTRACT

The Supplementary Information (SI) is organized as follows. Section 1 describes in detail how to compute optimal spatial shifts from epidemic snapshots using linear programming, and also discusses the flexibility of modelling shift costs. Section 2 provides additional descriptions of the pandemic timeline, COVID-19 data and Twitter movement data. Section 3 complements some findings of the main document with additional figures.

List of Figures

S1 A simple schematic representation for calculating the optimal spatial shifts between two epidemic snapshots. . . 2
S2 The temporal variation of total confirmed cases for eight selected states. 7
S3 The rank-size distributions of new confirmed cases in six pandemic phase. 8
S4 Flow maps of aggregated Twitter movements in six pandemic phases. 9
S5 Box plot of aggregated Twitter movements. 10
S6 States with larger GDP tend to have significantly stronger spatial shifts. 11
S7 Temporal changes of inferred spatial shifts with respect to total intensity and mean distance. 12
S8 Supplementary correlation analysis between spatial shifts and new confirmed cases. 13

List of Tables

S1 Key events to determine the pandemic phases 6

1. Methods and Models

Note 1: Calculating the optimal spatial shifts between snapshots

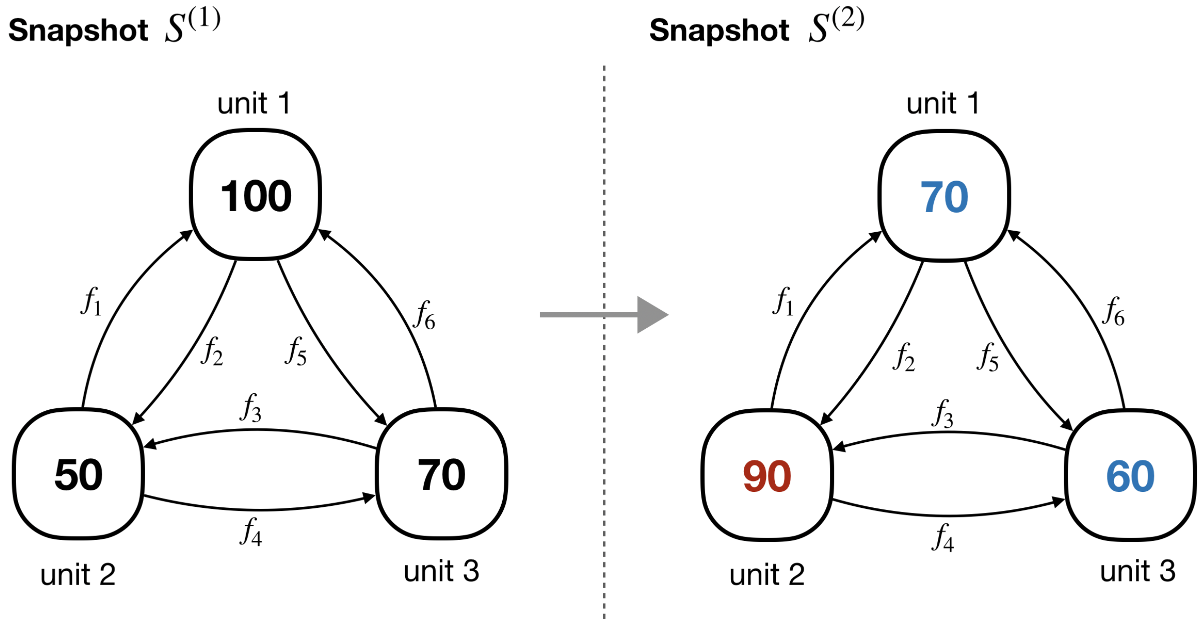

Figure S1. A simple schematic representation for calculating the optimal spatial shifts between two epidemic snapshots. f_1 and f_2 are the shifts between units 1 and 2, f_3 and f_4 are the shifts between units 2 and 3, f_5 and f_6 are the flows between units 1 and 3, respectively. The observed confirmed cases of unit 1 decreases from 100 to 70, unit 2 increases from 50 to 90 and unit 3 decreases from 70 to 60. The shift costs are assumed to be equal.

The strategy of inferring the spatial shifts of pandemic's spatial centres is similar to the minimum-cost flow problem in network optimization[1,2], which aims at finding the minimum-cost flow configuration (of shifts) that is subject to the variations (new confirmed cases) at all nodes (states).

Considering a simple schematic representation of our task illustrated in Fig. S1, where only three spatial units are included, we describe how the calculation is performed. Here, snapshot $S^{(1)}$ captures the case numbers distribution at time t_1 while Snapshot $S^{(2)}$ captures that of time t_2. f_i ($i \in [1,6]$) is the intensity of in-shifts or out-shifts from one spatial unit to another during the given time period. During t_2 and t_1, all potential shifts among the three spatial units can be represented by a single vector $\mathbf{f} = [f_1, f_2, f_3, f_4, f_5, f_6]$, where $\forall f_i \in \mathbf{f}$ we require $f_i \geq 0$. Assuming the unit shift cost is one, we have the following linear program:

$$
\begin{aligned}
\textit{minimize} \quad & f_1 + f_2 + f_3 + f_4 + f_5 + f_6 \\
\textit{subject to} \quad & -f_1 + f_2 - f_5 + f_6 = -30 \\
& f_1 - f_2 - f_3 + f_4 = 40 \\
& +f_3 - f_4 + f_5 - f_6 = -10 \\
& f_i \geqslant 0 \quad for \quad i = 1, 2, \ldots, 6,
\end{aligned}
\tag{1}
$$

where a minus sign denotes out-shift and a plus sign indicates in-shift. Each constraint equation ensures that the in and out-shifts is in consistent with the observed variation at a spatial unit. With an objective function of minimization, the optimal spatial shifts can well describe how the observed case variations are caused.

In Eq. 1, the matrix $A = \begin{pmatrix} -1 & 1 & 0 & 0 & -1 & 1 \\ 1 & -1 & -1 & 1 & 0 & 0 \\ 0 & 0 & 1 & -1 & 1 & -1 \end{pmatrix}$ represents the coefficients of all shift variables, while the observed variation can be described as a vector $\mathbf{b}^T = [-30, 40, 10]$. We then transform the linear program (Eq. 1) into equation

form[3], so that it can be directly solved by the simplex method[4,5]. By definition, the equation form is written as follows:

$$\begin{aligned} &\textit{minimize} && \mathbf{c}^T\mathbf{x} \\ &\textit{subject to} && A^{re}\mathbf{x} = \mathbf{b}^{re} \\ & && \mathbf{x} \geqslant \mathbf{0}, \end{aligned} \tag{2}$$

where $\mathbf{x}$ is a vector of n variables, and A^{re} is an $m \times n$ reduced row echelon form matrix with m rows and n columns ($n \geqslant m$) of rank m. $\mathbf{c} \in \mathbb{R}^n$, $\mathbf{b}^{re} \in \mathbb{R}^m$ and $\mathbf{0}$ is the zero vector with n components.

For a subset $B \subseteq \{1, 2, ..., n\}$, we let A_B denote the matrix consisting of the columns of A whose indices belong to B. For instance, for $A^{re} = \begin{pmatrix} 1 & -1 & 0 & 0 & 1 & -1 \\ 0 & 0 & 1 & -1 & 1 & -1 \end{pmatrix}$ and $B = \{1, 6\}$, we have $A^{re}_B = \begin{pmatrix} 1 & -1 \\ 0 & -1 \end{pmatrix}$. Thus, a basic feasible solution of Eq. 2 is an $\mathbf{x} \in \mathbb{R}^n$ for which there exists a set $B \subseteq \{1, 2, ..., n\}$ with m elements such that:

- the (square) matrix A^{re}_B is nonsingular, i.e., the columns indexed by B are linearly independent, and
- $x_j = 0$ for all $j \notin B$.

After Gauss-Jordan elimination[6], we can then convert the coefficient matrix A in linear program (1) into its reduced row echelon form $A^{re} = \begin{pmatrix} 1 & -1 & 0 & 0 & 1 & -1 \\ 0 & 0 & 1 & -1 & 1 & -1 \end{pmatrix}$, where A^{re} is a matrix of rank 2 with 2 rows and 6 columns, and the corresponding $\mathbf{b}^{re}$ of $\mathbf{b}$ is $[30, -10]$. Given $\mathbf{c} = (1, 1, 1, 1, 1, 1)$, the equation form of linear program (1) is rewritten as

$$\begin{aligned} &\textit{minimize} && f_1 + f_2 + f_3 + f_4 + f_5 + f_6 \\ &\textit{subject to} && f_1 - f_2 \qquad\qquad + f_5 - f_6 \quad = 30 \\ & && \qquad\qquad + f_3 - f_4 + f_5 - f_6 \quad = -10 \\ & && f_i \geqslant 0 \quad for \quad i = 1, 2, ..., 6. \end{aligned} \tag{3}$$

With the simplex method, we first reshape the linear program in the form of a *simplex tableau*. In the case of Eq. 3, we begin with $B = \{1, 6\}$, and the tableau is

$$\begin{array}{l} f_1 = 30 + f_6 \\ f_6 = 10 \\ \hline z = f_1 + f_6. \end{array} \tag{4}$$

The first two rows consist of equations of the linear program, in which the non-zero variables are on the left-hand side and the remaining terms are on the right-hand side.The last row separated by a line contains a new variable z, which represents the objective function. From this tableau, we obtain a feasible solution $\mathbf{f} = [40, 0, 0, 0, 0, 10]$ and $z = 50$, which is basic with $B = \{1, 6\}$.

Starting from the initial simplex tableau, we continue constructing a sequence of tableaus of similar form by gradually rewriting them until the optimal z is obtained. Due to the non-negativity constraint of $\mathbf{f}$, possible subsets $B \subseteq \{1, 2, ..., 6\}$ are $\{1, 4\}, \{1, 6\}, \{4, 5\}, \{5, 6\}$. Finally, we can obtain the optimal feasible solution with $z = 40$, $B = \{1, 4\}$ and $\mathbf{f} = [30, 0, 0, 10, 0, 0]$ when the simplex tableau is

$$\begin{array}{l} f_1 = 30 \\ f_4 = 10 \\ \hline z = f_1 + f_4. \end{array} \tag{5}$$

The previous example displays how to construct and calculate the optimal spatial shifts between distribution snapshots using the simplex method. Based upon that, we start to construct a generalized linear program for similar scenarios. Considering a study area consists of a set $\mathbf{N}$ with n spatial units (states in this work), we formalize the data of total COVID-19 confirmed cases in two consecutive epidemic snapshots, $S^{(t_1)}$ at time t_1 and $S^{(t_2)}$ at time t_2 (t_1 earlier than t_2) as:

$$\begin{aligned} D^{(t_1)} &= [d_1^{(t_1)}, d_2^{(t_1)}, ..., d_i^{(t_1)}, ..., d_{n-1}^{(t_1)}, d_n^{(t_1)}] \\ D^{(t_2)} &= [d_1^{(t_2)}, d_2^{(t_2)}, ..., d_i^{(t_2)}, ..., d_{n-1}^{(t_2)}, d_n^{(t_2)}], \end{aligned} \tag{6}$$

where $d_i^{(t_1)}$ and $d_i^{(t_2)}$ are the reported total confirmed cases in state $n_i \in \mathbf{N}$ by time t_1 and t_2, respectively. Since $d_i^{(t_1)} < d_i^{(t_2)}$ applies for all states at all time, we define $d_i^{(t_1)'} = d_i^{(t_1)} \sum_i d_i^{(t_2)} / \sum_i d_i^{(t_1)}$ as the rescaled number of $d_i^{(t_1)}$. The virtual variation of confirmed case at state n_i is $\Delta cc_i = d_i^{(t_2)} - d_i^{(t_1)'}$, ensuring a closed and static regional system with $\sum_i d_i^{(t_1)'} = \sum_i d_i^{(t_2)}$.

We use cost matrix $C \in \mathbb{R}^{n \times n}$ to describe the shifts' costs between t_1 and t_2, where $c_{i,j} \in C$ is the cost of shift from state n_i to n_j.

$$C = \begin{pmatrix} 0 & c_{1,2} & \cdots & c_{1,n-1} & c_{1,n} \\ c_{2,1} & 0 & \cdots & \cdots & \cdots \\ \vdots & \vdots & \vdots & \vdots & \vdots \\ \cdots & \cdots & c_{i,j} & \cdots & \cdots \\ \vdots & \vdots & \vdots & \vdots & \vdots \\ c_{n-1,1} & \cdots & \cdots & \cdots & \cdots \\ c_{n,1} & \cdots & \cdots & \cdots & 0 \end{pmatrix} \tag{7}$$

Also, we assume a fully-connected shift matrix $X \in \mathbb{R}^{n \times n}$, where $x_{i,j} \in X$ is the spatial shift variable from state n_i to n_j to be calculated.

$$X = \begin{pmatrix} 0 & x_{1,2} & \cdots & x_{1,n-1} & x_{1,n} \\ x_{2,1} & 0 & \cdots & \cdots & \cdots \\ \vdots & \vdots & \vdots & \vdots & \vdots \\ \cdots & \cdots & x_{i,j} & \cdots & \cdots \\ \vdots & \vdots & \vdots & \vdots & \vdots \\ x_{n-1,1} & \cdots & \cdots & \cdots & \cdots \\ x_{n,1} & \cdots & \cdots & \cdots & 0 \end{pmatrix} \tag{8}$$

Then, similar to a previous work of the authors[7], the generalized linear program for inferring spatial shifts can be constructed as follows:

$$\begin{aligned} \textit{minimize} \quad & C^T \times X \\ \textit{subject to} \quad & -\sum_{j \in \mathbf{N}} x_{i,j} + \sum_{j \in \mathbf{N}} x_{j,i} = \Delta cc_i, \quad \forall i \in \mathbf{N} \\ & x_{i,j} \in \mathbb{R}, \quad x_{i,j} \geq 0 \quad \forall i,j \in \mathbf{N} \end{aligned} \tag{9}$$

Because a linear program in the form of Eq. 9 can always be transformed into the equation form (2), we can always find an optimal solution for X using the simplex method as demonstrated before, if the optimal basic solution does exist. In the case of COVID-19 epidemic snapshots, the meaning of inferred X would be the shifts of pandemic's spatial centres with respect to the number of COVID-19 confirmed cases. The optimization process in this work was implemented using *PuLP*, which is an open Python package for linear programming (https://coin-or.github.io/pulp). All code and data needed to replicate this research will be available at https://github.com/dizhu-gis/CovIDSpatialShifts once the paper is published.

Note 2: Modelling the cost of spatial shifts

The concept of spatial shift in this study is similar to that of spatial interaction, which could also be considered as a second-order spatial measure between two geographic units or objects. In practice, the scale of a spatial interaction may be measured by physical phenomena such as the volumes of passengers, migration flows, trade flows, currency flows among spatial units. By extension, the intensity of a spatial shift can be measured by the virtual shift of geographic attributes such as COVID-19 case number in two spatial distributions.

In literature, the modelling of aggregated[8] and individual[9–11] spatial interactions can be viewed as two sides of the same coin[12], the intensities of which are governed by the distance decay effect and are influenced by the nodal attractions of locations[13]. Spatial interactions are often regarded as the driving force that transform the distributions of geographic attributes[14]. Derived from Newton's law of gravity, a simple gravity model to describe the relationship between spatial interactions and spatial distributions is written as:

$$F_{i,j} = \alpha A_i A_j / f(d_{i,j}), \tag{10}$$

where $F_{i,j}$ is the potential spatial interaction from place i to j, A_i and A_j are the places' attractions (usually measured by population sizes), and $f(d_{i,j})$ is the distance decay function determined by the distance $d_{i,j}$ between two places. Eq. 10 could be also considered as an ideal representation of the spatial shift. The distance decay function $f(d_{i,j})$ may have several forms according to various applications, among which the power function d^β is most widely used in regional studies[7,12,15]. The parameter β reveals the effect of distance decay: a greater β indicates a stronger distance decay effect such that spatial shifts are more likely to be impeded due to the geographic segregation.

Inspired by the prevailing gravity-based spatial interaction models, the unit cost of spatial shifts from place i to j can be modelled as a reversed form of Eq. 10, i.e., $c_{i,j} = k\frac{d^\beta}{A_i A_j}$, where $k = 1/\alpha$. Noting that the total cost of all the shifts in the system is assumed to be a constant $C_0 = \sum_i \sum_j c_{i,j} X_{i,j}$ when the evolutionary aim of the regional system is an entropy optimization of the shift configuration[8]. Meanwhile, when we adopt a gravity-based form of $X_{i,j} \propto \frac{A_i A_j}{d^\beta}$, the total cost of shifts among all spatial units $\sum_i \sum_j c_{i,j} X_{i,j}$ would remain fixed. This definition of shifts' unit cost leads to a reasonable curve for the distance decay of shift costs which is convex and non-linear,

$$\frac{\partial c}{\partial d} = \beta d^{\beta - 1}, \tag{11}$$

indicating that the unit cost of spatial shifts increases, but at a sub-linear rate with distance, when $\beta < 1$.

To consider COVID-19 scenarios, we add an extra term to characterize the social distancing effect on spatial shifts, i.e., $T_{i,j} = log_{10}(m_{i,j} + \delta)$ where $m_{i,j}$ refers to the aggregated twitter movements from state n_i to n_j. Logarithmic transformation is applied to $m_{i,j}$ to reduce the skewness of data distribution for twitter movements and $\delta = 1$ is a threshold parameter to avoid the zero value of $m_{i,j}$. This term adds more information about the dynamic mobility restrictions during the pandemic so that the spatial shifts are calculated based on hybrid constraints of both geographic distancing and social distancing.

To this end, we define the unit cost of pandemic spatial shifts in this work as:

$$c_{i,j} \propto \frac{G_{i,j}}{T_{i,j}} = \frac{k\frac{d_{i,j}^\beta}{A_i A_j}}{log_{10}(m_{i,j} + \delta)}. \tag{12}$$

Taking this form of cost into Eq. 9, it is easy to tell that the coefficient k does not affect our calculation. In the main document, we present the results using census resident population as As, $k = 10^8$, $\delta = 1$, and a distance decay parameter $\beta = 0.8$. Since the authors have discussed the influence of different βs and different As on the inferred results in a previous work[7], we think it is beyond the scope of this empirical study. Interested readers could refer to[7] for more details. It is necessary to admit that our modelling of spatial shift cost can be improved, as there can be better ways to determine the distance decay function $f(d_{i,j})$[16] and nodal attractions A depending on context and available data. The key point here, however, is the idea to derive spatial shift costs based on certain spatial interaction models, such that the inference of spatial shifts in Eq. 9 could contain some meaningful geographical and social concerns.

2. Data

Timeline measures of pandemic phases

Some important dates during the timeline between Jan. 21, 2020 and Aug. 09, 2020 are selected to help determine the six interested pandemic phases in our research. These COVID-19 related events are publicly reported in news and are collected from the internet[17]. Other ways of dividing the timeline are also acceptable based on different research goals.

Table S1. Key events to determine the interested pandemic phases in this study.

Date	Events	Phase
2020. 1. 21	Officials in Washington state confirm the first case on US soil.	N/A
2020. 1. 30	The United States reports its first confirmed case of person-to-person transmission of the coronavirus. WHO determines that the outbreak constitutes a PublicHealth Emergency of International Concern (PHEIC).	N/A
2020. 1. 31	The U.S. government announces it will deny entry to foreign nationals who have traveled in China in the last 14 days.	P1
2020. 2. 11	WHO names the coronavirus Covid-19.	
2020. 3. 11	WHO declares the novel coronavirus outbreak to be a pandemic. The U.S is restricting travel from Europe to slow the spread of coronavirus.	
2020. 3. 13	The U.S. declares a national emergency to free up $50 billion in federal resources to combat coronavirus.	P2
2020. 3. 31	Most states have reacted to the stay-at-home order.	P3
2020. 4. 8	China reopens Wuhan after a 76-day lockdown.	
2020. 4. 28	The total number of confirmed cases reaches one million.	P4
2020. 5. 25	The death of George Floyd sparked civil right protests and anti-lock down protests across the United States.	
2020. 6. 11	The total number of confirmed cases reaches two million.	P5
2020. 7. 7	The U.S. administration notifies Congress and the UN that the US is formally withdrawing from WHO.	
2020. 7. 23	The total number of confirmed cases reaches four million.	P6
2020. 8. 09	The total number of confirmed cases reaches five million.	

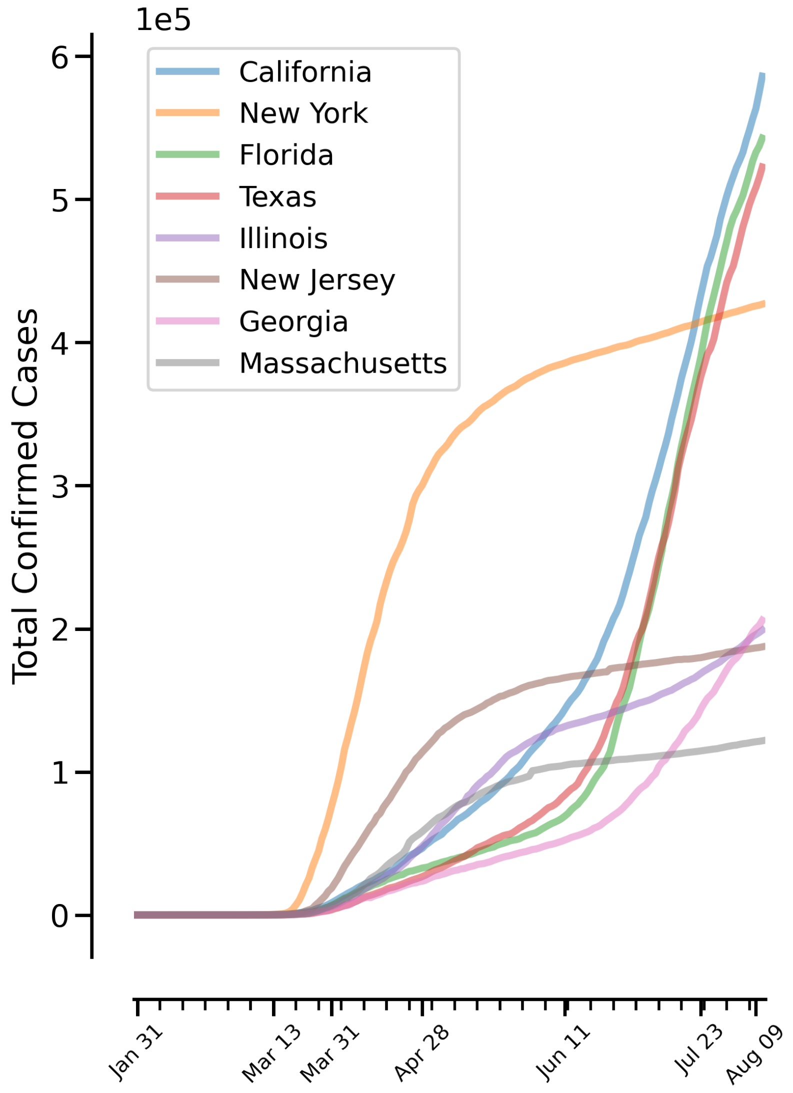

Figure S2. The temporal variation of total confirmed cases for eight selected states. The daily reported number of cumulative confirmed cases is collected for all the states in this study. We only visualize the curves for eight representative states in this figure. Compared to the early outbreak of New York, delayed but similar trends can be identified in states such as New Jersey and Massachusetts.

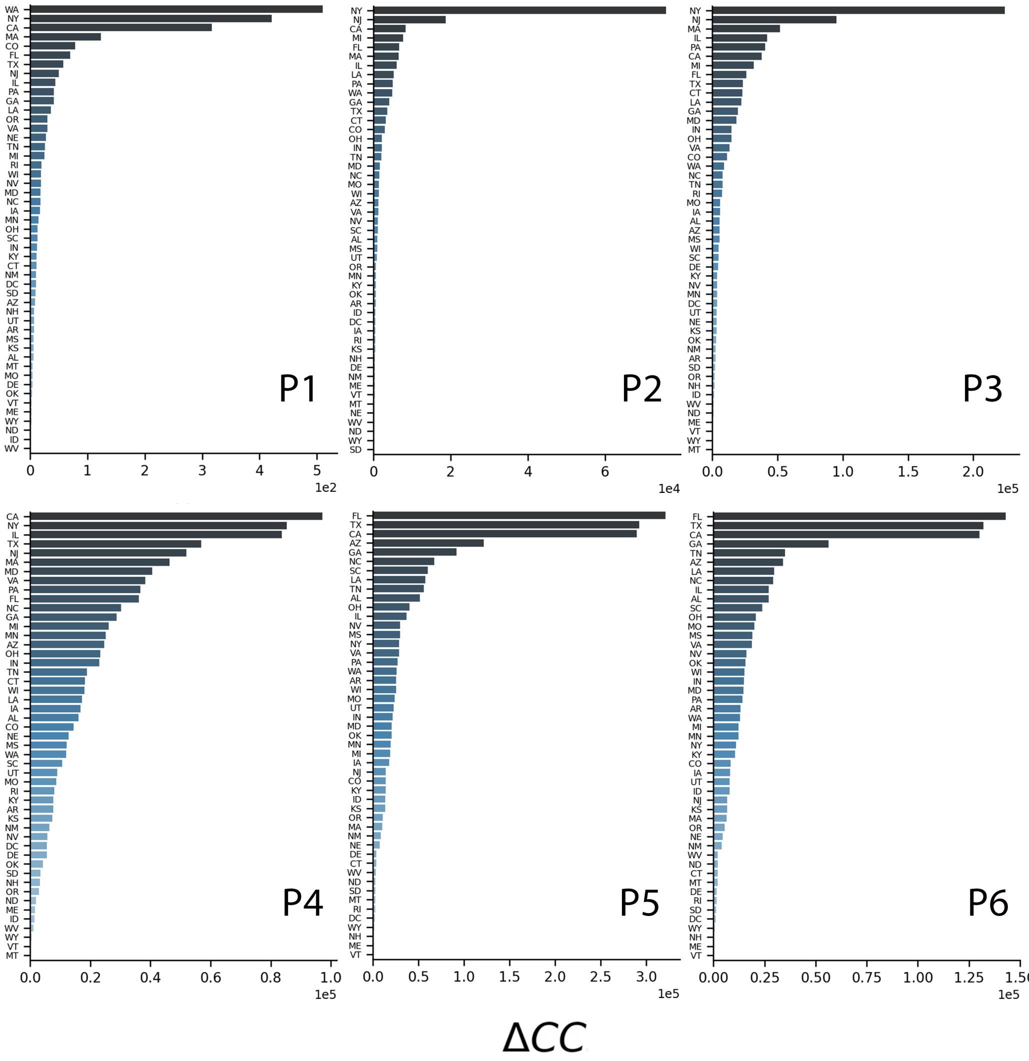

Figure S3. The rank-size distributions of new confirmed cases in six pandemic phase. Based on these rank-size distributions, we are able tell the absolute variation of each state. These numbers of new confirmed cases act as the input Δcc_i of our optimization model (Eq.9) to infer the pandemic spatial shifts.

Twitter movement data

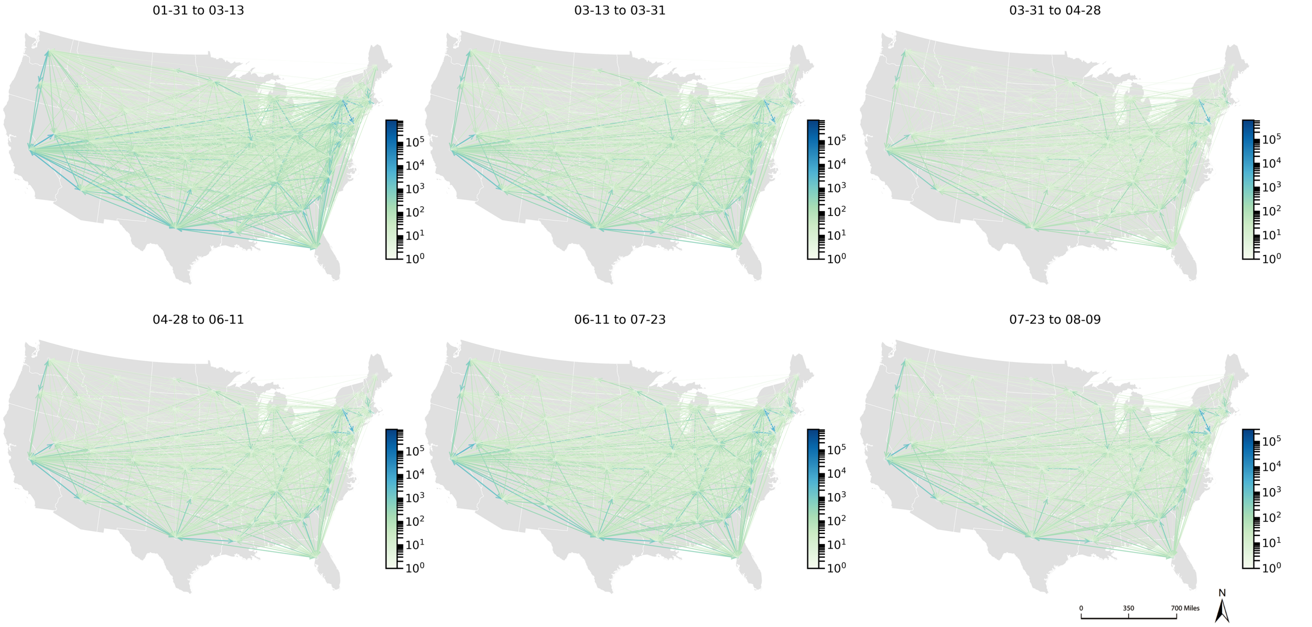

Figure S4. Flow maps of aggregated Twitter movements in six pandemic phases. Movements are drawn as arrows among states' centroids indicating where people come from and where they move to. The colours and widths of all arrows are linearly mapped according to the number of movements.

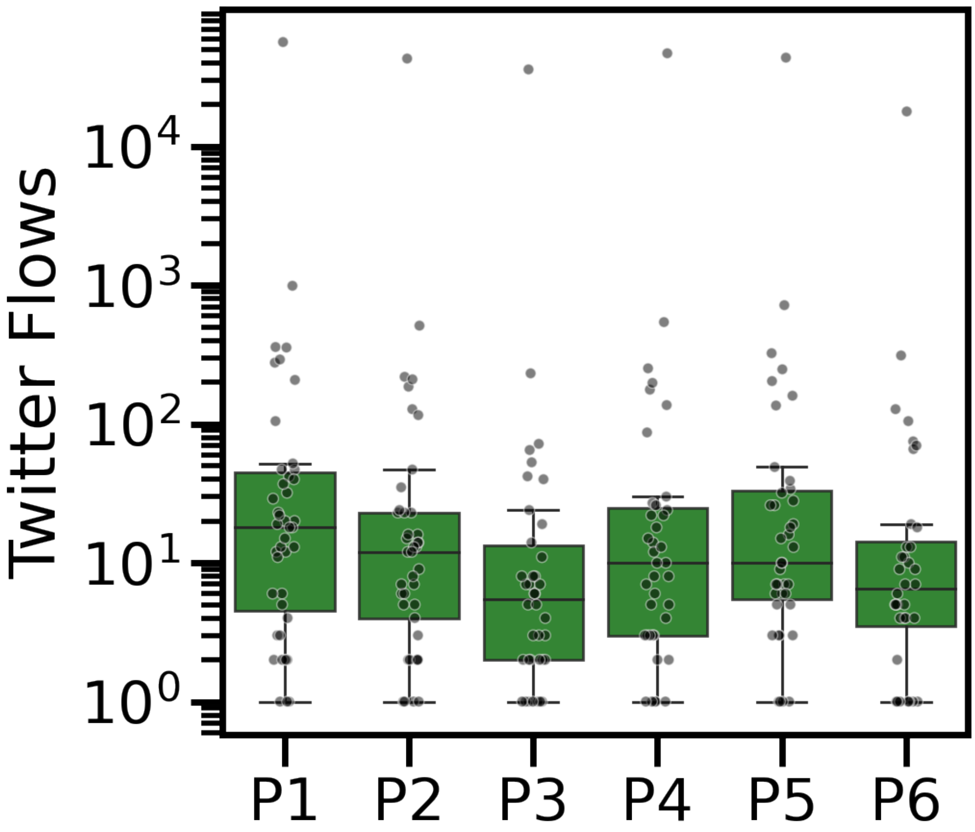

Figure S5. Box plot of aggregated Twitter movements. Each black dot denotes the intensity of a pair-wise aggregated flow between two states during a certain phase. The horizontal line in the box shows the median intensity of all flows. The box plot indicates the dynamic situation of mobility restrictions in six phases, as we can see the obvious decline of human movements during the earlier phases such as P2 and P3.

3. Supplementary results

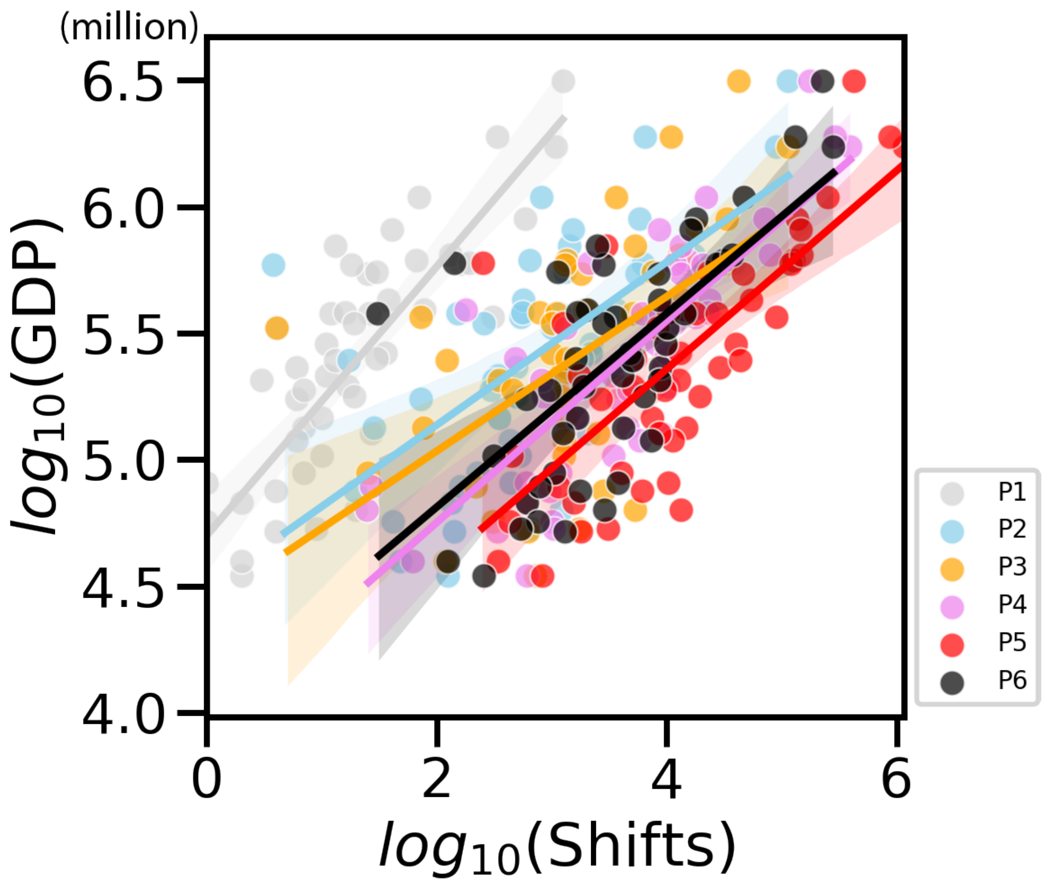

Figure S6. States with larger GDP tend to have significantly stronger spatial shifts. The Pearson coefficients in the six phases are $R_1 = 0.82$, $R_2 = 0.63$, $R_3 = 0.56$, $R_4 = 0.80$, $R_5 = 0.74$ and $R_6 = 0.67$, respectively (with all p-values ≈ 0).

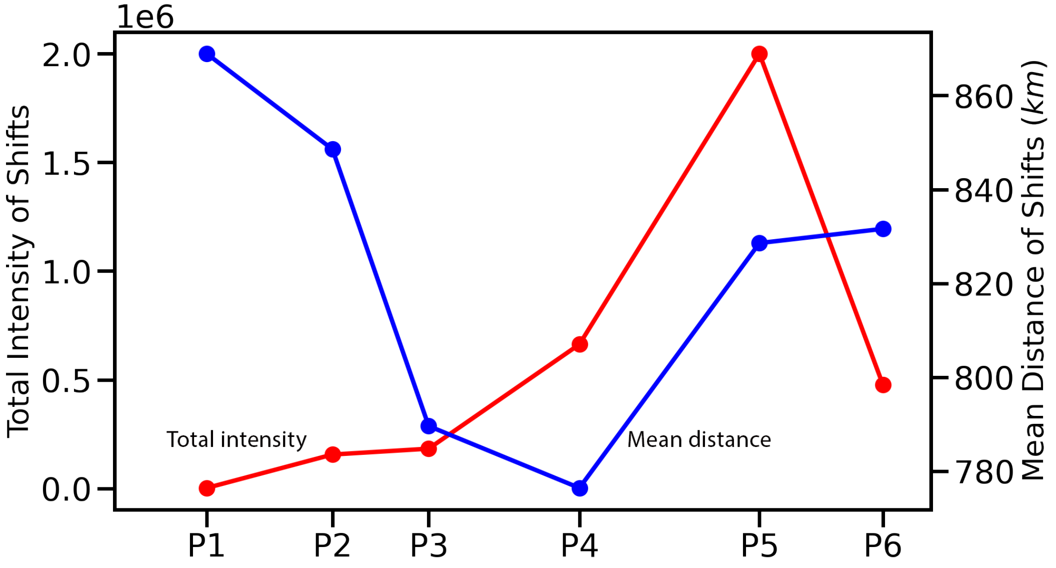

Figure S7. Temporal changes of inferred spatial shifts with respect to total intensity (red) and mean distance (blue) reflect how the pandemic develops and shifts across six phases.

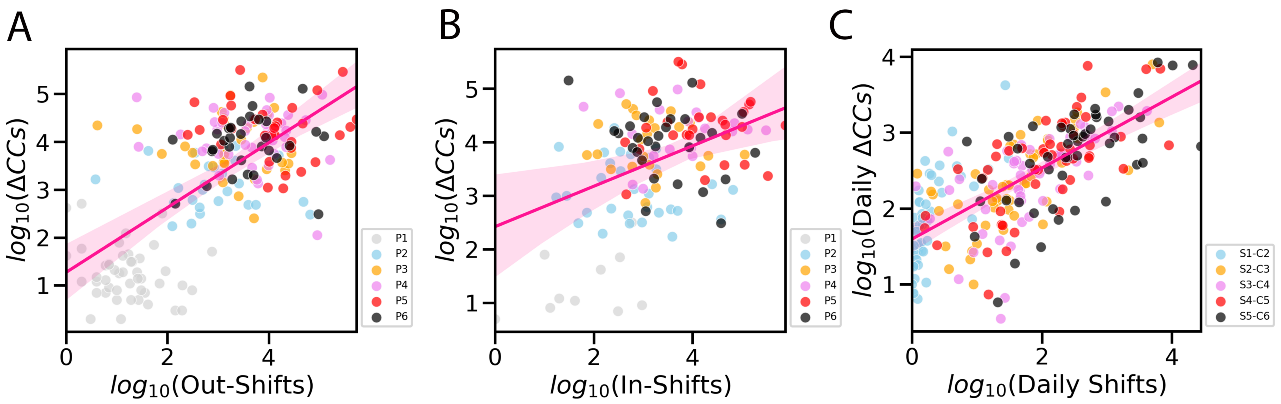

Figure S8. Supplementary correlation analysis between spatial shifts and new confirmed cases. Significant positive correlations are found between (A) $log_{10}(\Delta CCs)$ and log_{10}(Out-Shifts) (Pearson: $R = 0.68$, $p \approx 0$), (B) $log_{10}(\Delta CCs)$ and log_{10}(In-Shifts) (Pearson: $R = 0.42$, $p \approx 0$) and (C) the cross-phase relationships (S_t-C_{t+1}) between states' daily shifts in a previous phase and their daily ΔCCs in the next phase (Pearson: $R = 0.72$, $p \approx 0$; Spearman: $R = 0.75$, $p \approx 0$).

References

1. Ahyja, R. K., Orlin, J. B. & Magnanti, T. L. *Network flows: theory, algorithms, and applications* (Prentice-Hall, 1993).
2. Cook, W., Lovász, L., Seymour, P. D. *et al. Combinatorial optimization: papers from the DIMACS Special Year*, vol. 20 (American Mathematical Soc., 1995).
3. Matousek, J. & Gärtner, B. *Understanding and using linear programming* (Springer Science and Business Media, 2007).
4. Nelder, J. A. & Mead, R. A simplex method for function minimization. The computer journal **7**, 308–313 (1965).
5. Dantzig, G. B. & Thapa, M. N. *The simplex method* (Springer, 1997).
6. Leon, S. J. *Linear algebra with applications* (Macmillan New York, 1980).
7. Zhu, D., Huang, Z., Shi, L., Wu, L. & Liu, Y. Inferring spatial interaction patterns from sequential snapshots of spatial distributions. International Journal of Geographical Information Science **32**, 783–805 (2018).
8. Wilson, A. G. *Entropy in urban and regional modelling*, vol. 1 (Routledge, 2011).
9. Barabási, A. L. The origin of bursts and heavy tails in human dynamics. Nature **435**, 207–11 (2005).
10. Han, X. P., Wang, B. H. & Zhou, T. Researches of human dynamics. Complex Systems and Complexity Science **7**, 132–144 (2010).
11. Song, C., Koren, T., Wang, P. & Barabási, A.-L. Modelling the scaling properties of human mobility. Nature Physics **6**, 818–823 (2010).
12. Liu, Y., Sui, Z., Kang, C. & Gao, Y. Uncovering patterns of inter-urban trip and spatial interaction from social media check-in data. PloS one **9**, e86026 (2014).
13. Miller, H. J. Tobler's first law and spatial analysis. Annals of the Association of American Geographers **94**, 284–289 (2004).
14. Ratti, C. *et al.* Redrawing the map of great britain from a network of human interactions. PLoS ONE **5**, e14248 (2010).
15. Wang, F. Measurement, optimization, and impact of health care accessibility: a methodological review. Annals of the Association of American Geographers **102**, 1104–1112 (2012).
16. Chen, Y. The distance-decay function of geographical gravity model: Power law or exponential law? Chaos Solitons and Fractals the Interdisciplinary Journal of Nonlinear Science and Nonequilibrium and Complex Phenomena **77**, 174–189 (2015).
17. CNN Editorial Research, Coronavirus Outbreak Timeline Fast Facts. https://www.cnn.com/2020/02/06/health/wuhan-coronavirus-timeline-fast-facts/index.html (2020).
